# Venous thromboembolism prevention post neck of femur fractures – does it make a difference?

**DOI:** 10.1186/1477-9560-6-8

**Published:** 2008-06-26

**Authors:** Radwane Faroug, Shireesha Konnuru, San S Min, Fazleenah Hussain, George Ampat

**Affiliations:** 1 Department of trauma and orthopaedics, Southport and Ormskirk Hospital NHS trust, Southport, UK

## Abstract

Neck of femur fractures predispose patients to venous thromboembolism (VTE). NICE has issued guideline 46 to reduce this risk through the use of antithrombic agents. We audited our department's VTE practise by reviewing the clinical notes of 123 consecutive patients with no exclusions. We found our compliance to be a low 6%. We also found that patients were likely to be given low molecular heparin (LMWH) only during their hospital stay. Reasons for the low adherence were probably secondary to confusion caused by the multiple thromboprophylaxis protocols used in our department. The correlation between duration of heparin administration and length of hospital stay was due to logistical difficulty in administering VTE prophylaxis out of hospital setting.

## Background

In the absence of thromboprophylaxis the risk of developing a DVT post a primary total hip replacement is 50% and 60% post a primary total knee replacement. The risk of the DVT progressing to a PE is 1% and the risk of the PE being fatal is 0.1%, a cumulative risk of fatality of 1 in 200 000 [1]. The mortality from a PE however increases markedly to 12.9% [2] in the neck of femur fracture (NOF) population not given prophylaxis. This increase being attributed to an older patient population with multiple co-morbidities, undergoing emergency surgery.

A meta analysis of randomised clinical trials has illustrated the progressive decrease in the incidence of DVT post total hip and knee replacement with increasing sophistication of anti-thrombic agent. For total hip replacement without prophylaxis 50% will develop a DVT, with aspirin 41.7%, adjusted dose warfarin 22.3%, LMWH 14.8%, and the rate being lowest with fondaparinux at 4.7%. A similar trend is present following total knee replacement. The incidence of DVT is 60% without prophylaxis, 54.6% with aspirin, 37% with adjusted dose warfarin, 35.1% with LMWH, and 12.5% with fondaparinux [3].

To reduce the risk of venous thromboembolism (VTE) in inpatients undergoing surgery – NICE issued clinical guideline 46 and The National Collaborating Centre for Acute Care published by the Royal College of Surgeons of England (RCSENG – NCC – AC) also published its guideline on VTE prevention in 2007. Both stated that patients having surgery for hip fracture should be offered mechanical prophylaxis and either LMWH or fondaparinux. LMWH or fondaparinux therapy should be continued for 4 weeks after hip fracture surgery.

In November 2007 we audited our Trauma department's VTE prevention practice.

## Objectives of audit

To measure our department's compliance with NICE guideline CG046

To investigate the incidence of DVT/PE

To investigate the causes of non compliance

To offer conclusions and recommendations

## Method

123 consecutive patients with no exclusions, who sustained a neck of femur fracture (NOF) (intra- or extra-capsular) between April and November 2007, and who were treated by the Southport and Ormskirk Trauma and Orthopaedic department were included in this audit.

## Parameters measured

1. Whether low molecular weight heparin (LMWH) was given for four weeks post surgery

2. The actual period of time for which LMWH was given post surgery

3. Whether any of the patients developed a DVT or PE prior to discharge

## Results

Of the 123 patients included in the audit, 8 died in hospital. Only one of the deaths was due to CTPA proven pulmonary embolism. Another patient developed uncomplicated deep venous thrombosis. An overall rate of VTE of 1.7%.

In the remaining 115 patients, LMWH was given for 4 weeks post surgery to 7(6%) patients only. There were 34(29%) cases where LMWH was not given for any period of time. Reasons why LMWH was not given were as follows: 1 case of haematuria, 9 cases where the patients were already on aspirin, 1 case where the patient was taking clopidogrel, 2 patients were on warfarin, and 1 patient refused. In 20 cases the reason for omission was not stated.

It was interesting to note that where LMWH was given, the period of administration corresponded to patient length of stay in 85% of cases. The mean duration of LMWH administration in NOF patients was 9.2 days. This implies that most of the patients treated with LMWH were treated whilst they were inpatients only.

## Conclusion

The compliance in our Trauma department with NICE guideline CG046 is low. It was implemented in 6% of cases only.

Due to logistical reasons, LMWH tends to be given for the duration of the patient's length of stay only. Methods to facilitate the administration of LMWH for four weeks post NOF surgery can include training the patient or relative in subcutaneous injection of LMWH, or arranging district nurse follow up. It also appears that in 10 (8.7%) of cases aspirin and clopidogrel were wrongly assumed to be alternatives to LMWH.

Interviews with the nursing staff highlighted the high number of differing thromboprophylaxis protocols currently in use in our Trauma department as a possible cause of confusion and low compliance.

## Recommendations and discussion

The Trauma and Orthopaedic department should adopt a single thromboembolism prevention protocol and follow the evidence based NICE and RCSENG – NCC – AC guidelines. Junior Doctors, Nurses and Pharmacy staff should be educated in the content of the above guidelines and the significance and incidence of VTE post NOF surgery.

A laminated copy should be provided in a prominent place on the ward as an aide memoire. Following the introduction of these recommendations, the data must be re-audited to make sure that the compliance has increased.

The importance of providing thromboprophylaxis for four weeks post NOF surgery should be stressed. Following TKR, THR or NOF surgery, there is a spike in hypercoagulability in the immediate post operative period with gradual decay over time. However for THR and NOF patients there is a bimodal distribution with a second peak in VTE incidence at three to four weeks post operatively [4].

It is interesting to read in the National Joint Registry 4^th ^annual report [5] that for primary hip replacement surgery, mortality rates up to 1 year were compared between patients with different types of thromboprophylaxis prescribed at the time of surgery including those given no prophylaxis. The evidence showed that there was no statistically significant difference in mortality between the different types of prophylaxis prescribed including no prophylaxis.

It is however our opinion that we cannot extrapolate these findings to include the NOF population until specific evidence becomes available.
